# Influence of Ceramic Material Type and Cement Shade on the Translucency of Lithium Disilicate Ceramic Veneers

**DOI:** 10.1155/2024/2540174

**Published:** 2024-11-15

**Authors:** Ali Dhahee Malallah, Nadia H. Hasan, Mohammed Hazim Qasim

**Affiliations:** Department of Conservative Dentistry, College of Dentistry, University of Mosul, Mosul, Iraq

**Keywords:** e.max CAD, e.max Press, laminate veneer

## Abstract

**Aims:** Careful selection of materials and resin cement shade can minimize color changes in laminate veneers. This study aimed to evaluate the influence of two ceramic material types, lithium disilicate glass–ceramic material (Ivoclar, Schaan/Liechtenstein; IPS e.max), computer aided design (CAD) and IPS e.max Press, and four different resin cement shades on color changes in lithium disilicate ceramic laminate veneers.

**Methods:** Forty extracted human maxillary first premolars were prepared to receive a laminate veneer. Optical scanning and digital designing were used to prepare 20 IPS e.max CAD and 20 IPS e.max Press veneer samples. These samples were divided into four groups based on resin cement shade and material: Group CAD A1 : (IPS e.max CAD with A1 cement shade), Group CAD T : (IPS e.max CAD with translucent cement shade), Group CAD M : (IPS e.max CAD with milky bright cement shade), Group CAD B1 : (IPS e.max CAD with B1 cement shade) and the same divisions for IPS e.max Press. Before cementation, color measurements were obtained using precision colorimeter NR110. After cementation, the *∆E* (color change) value was recorded and tabulated. One-way analysis of variance (ANOVA) and Duncan's post hoc test were used to evaluate the influence of ceramic material types and cement shade on *∆E*.

**Results:** There were significant differences between the IPS e.max CAD and e.max Press veneers for the four tested cement shades. The highest color change was observed in press M veneers (8.84 ± 0.63931) while the lowest color change was observed in CAD M veneers (0.5 ± 0.16371). There were significant differences in color change based on cement shade, with B1 showing the greatest change (8.84 ± 0.47440 for Press veneers and 2.3 ± 0.11992 for CAD veneers).

**Conclusions:** Different shades of resin cements and different manufacturing techniques produce different levels of color changes therefore careful selection of materials and cement shade can minimize color changes in laminate veneers.

## 1. Introduction

Achieving high-quality patient outcomes can be challenging for dental practitioners and ceramists, particularly in case of ceramic veneers (CVs). Dental ceramics are used in dentistry to replace missing parts of teeth; restore traumatized, fractured, or worn dentition; and correct abnormal anatomy or mispositioned teeth. Additionally, dental ceramics can be used for teeth with moderate discoloration resulting from factors such as tetracycline, fluoride exposure, aging, or amelogenesis imperfecta [1, 2].

Dental ceramics have suitable properties for buildup indirect restorations that play a vital role in the success of dental restorations. They are used extensively owing to their desirable properties, including excellent biocompatibility, color stability, chemically inert, increased flexural strength, low thermal conductivity, radiopacity, and ability to replicate natural function and aesthetics [3, 4].

Lithium disilicate (enriched glass ceramic), which is strengthened by orthophosphate crystals in a glassy matrix, can be used in a pressed or milled form. This type of glass ceramic is used widely in the creation of laminate veneers due to its exceptional esthetic properties, as well as its sufficient strength and bonding capabilities, which make it suitable for thin veneers. By ensuring that the crystalline and glassy phases of lithium disilicate have a similar refractive index, it is possible to achieve a highly translucent composition of the material [5–7].

Several translucencies and shade ingots are available for the lithium disilicate glass–ceramic material (Ivoclar, Schaan/Liechtenstein; IPS e.max) Press and computer aided design (CAD) lithium disilicate ceramics (Ivoclar, Schaan, Liechtenstein) used in this study. Translucency refers to the property of permitting the passage of light and scattering it in a way so that a picture is obscured to a certain degree. Translucency lies between complete opacity and transparency [8].

The translucency of laminate veneers is influenced by various factors, including the type of material used and the shade of the luting cement. The chemical composition and polymerization reaction of resin luting cement, directly impact the esthetic result of ceramic restoration. As the color of the underlying tooth or veneering material can be masked by the resin cement, careful selection of the luting cement is essential for achieving optimal esthetic outcomes, particularly when using highly translucent materials for laminate veneers. In such cases, it is important to avoid using resin cement of a darker shade [9–11].

The success of CVs depends on several factors, including the color of the underlying tooth, thickness of the veneers, and the type of resin cement used. However, the challenge of shade selection and matching for veneers is further complicated by the shade of the luting agent, as cementation of the veneers can also affect the resulting shade. Therefore, studies have been conducted on the impact of different luting agents on the final shade of CVs, they were controversial in their results about the effect of cement shade and ceramic material on the color changes of the laminate veneer and the current study has proved the effect of the resin cement shade and ceramics on the color change of laminate veneers in addition the previous studies used different methodology and specimens types and preparations and the current study conducted on natural teeth instead of geometrical and resin dies specimens [1, 9, 10].

The current study aimed to evaluate the influence of ceramic material types, IPS e.max CAD and IPS e.max Press, and four different cement shades on color changes in lithium disilicate ceramic laminate veneers. The null hypothesis was that neither the ceramic material type nor the luting cement shade would have an effect on the color changes in laminate veneers.

## 2. Materials and Methods

Forty human intact maxillary first premolars freshly-extracted for orthodontic reasons were selected with the approval of the Research Ethics Committee (UoM.Dent/H.DM.45/22). The individuals provided informed consent regarding the collection of their teeth for this study. Teeth were cleaned and any adhering soft tissues, superficial stains, and calculus deposits were removed with a hand scaler.

**Figure 1:**
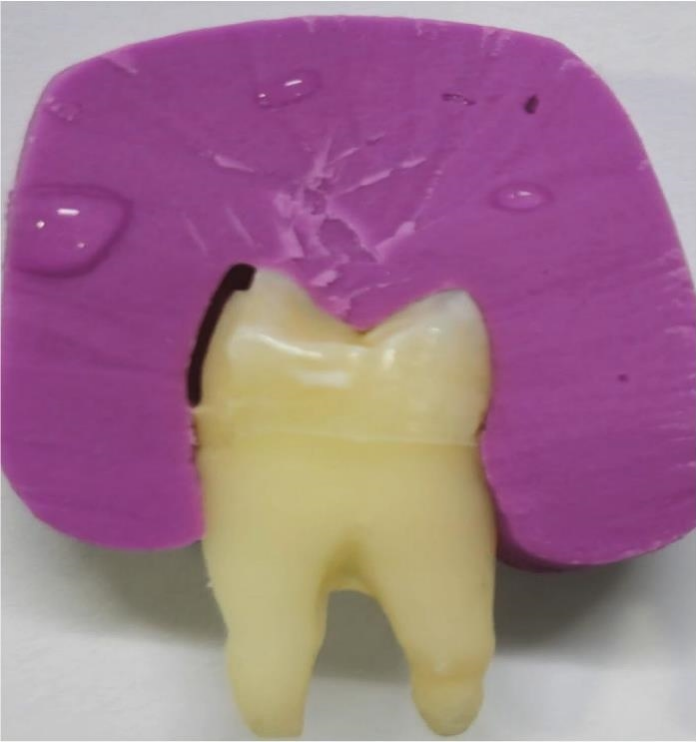
A fully prepared tooth for veneer with silicon index.

A silicon impression material index was made for each tooth before the preparation. Standardized preparation dimensions were created for each tooth using a three-wheel depth cutter diamond bur. The buccal surface reduction was 0.5 mm at the cervical third and 0.7 mm at the middle and occlusal thirds. The preparation extended 1 mm occlusal to the cemento-enamel junction, the buccal cusp reduction was 1.5 mm occluso-cervically and 1 mm bucco-palatally, and the margin was placed away from the occlusal contact, as shown in Figure 1 [12–15].

All specimens were randomly divided into eight groups (five samples in each group) according to the material type and cement shade:

  Group Press A1: (IPS e.max Press with A1 cement shade)
  Group Press T: (IPS e.max Press with translucent cement shade)
  Group Press M: (IPS e.max Press with milky bright cement shade)
  Group Press B1: (IPS e.max Press with B1 cement shade)
  Group CAD A1: (IPS e.max CAD with A1 cement shade)
  Group CAD T: (IPS e.max CAD with translucent cement shade)
  Group CAD M: (IPS e.max CAD with milky bright cement shade)
  Group CAD B1: (IPS e.max CAD with B1 cement shade)

**Figure 2:**
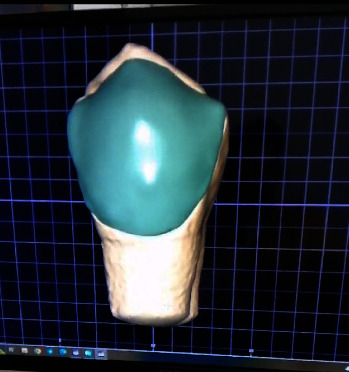
CV design for e.max CAD samples. CAD, computer aided design; CV, ceramic veneer.

Impressions were taken for each specimen (the 40 previously prepared teeth) using additional silicone and poured using type IV extra hard dental stone. Twenty of the stone dies were scanned using an optical scanner (Zirconzhan, s600arti, Italy) to obtain digital images for the prepared teeth for IPS e.max CAD samples. For IPS e.max CAD samples, 20 CVs were designed using an intelligent design software with cement thickness of 60 *μ* m, as shown in Figure 2.

Milling of the 20 laminate veneers from IPS e.max CAD low translucency (LT) blocks with shade A2 (Ivoclar, Schaan, Liechtenstein, Germany) was performed using an open milling machine according to the manufacturer's instructions. The milling was performed under continuous water cooling according to the manufacturer's instructions and then subjected to a crystallization cycle at 840°C using Programat 310 CS furnace (Ivoclar).

The other 20 stone dies were used to make 20 laminate veneer wax patterns for lithium disilicate IPS e.max Press samples that were pressed into IPS e.max Press lithium disilicate LT ingots with shade A2 (Ivoclar) using a press machine furnace (Ep 500 IPS Empress; Ivoclar) to complete the crystallization cycle and were stored in distilled water in preparation for the next step for color measurements and cementation.

### 2.1. Color Measurements

Color measurement of the samples was recorded in terms of L^*∗*^, a^*∗*^, and b^*∗*^ coordinates from the International Commission on illumination system (CIELAB, Vienna, Austria). Forty samples were tested, and the readings were recorded at two time points:

1. Before cementation: After preparation where the teeth prepared for CV, after removal from the storage water and gently dried and application of glycerin gel without luting cements and then restored in distilled water for measurement after cementation.
2. After cementation with luting cements (A1, transluscent, milky bright, and B1 shades).

Before cementation, the specimens were placed on their corresponding previously-prepared teeth without cementation and grouped according to the type of material and cement shade, as described previously.

**Figure 3:**
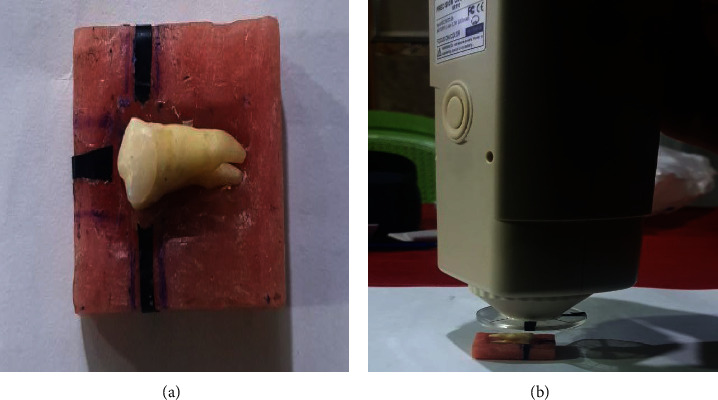
Color measurement of the samples. (a) Self-cure positioning guide for the samples; (b) color measurement of the sample using a calorimeter.

To ensure that the same area of the tooth was evaluated by the colorimeter every time, a “positioning guide” was fabricated from self-cure acrylic resin. The positioning guide was filled with microcrystalline wax in which the tooth was embedded. Three markings were placed on either side of the tooth and one in the center of tooth, as shown in Figure 3a [1].

To reduce the edge loss effect (light within the specimen scattered to the edges without being absorbed), a drop of glycerin was placed between each specimen and the backing to seal the airspace and ensure that the specimen was in optical contact with the backing. Glycerin was used in this study as its refraction index is similar to porcelain, minimizing the light refraction that occurs when the light beam crosses substrates with different refractive indices such as air and porcelain [16, 17].

All the specimens were tested for color reproduction using a handheld colorimeter (NR110 Precision colorimeter, Schenzhen 3nh technology, China). Color measurements were performed by positioning the aperture of the portable colorimeter (5 mm diameter) perpendicularly against the middle of the facial surface of the specimen, as shown in Figure 3b. The aperture of the colorimeter was in full contact with the surface of the veneer to determine the *∆E* of all the specimens. Three measurements were taken for each specimen. The colorimeter was calibrated after every two measurements to ensure standard reproducibility [1, 17].

For color measurement after cementation, the specimen were removed from water and surface treatment of each tooth was performed using 32% phosphoric acid etching gel (Unitech, Choice 2, Bisco, Chicago, IL, USA), which was applied on the enamel surface for 15 s according to manufacturer's instruction and then rinsed using air/water spray. Light-curing double bottle bonding agent (All-Bond3^R^; Choice 2, Bisco) was mixed and applied to the etched tooth surface and air-dried for 10 s and light-cured for 10 s according to manufacturer's instruction.

The intaglio surface of the IPS e.max laminate veneers was etched using hydrofluoric acid etching (9.5% porcelain etchant gel) for 90s, rinsed, thoroughly dried, and treated with silane agent (BIS-Silane; Choice 2, Bisco) using an application brush, leaving the primer for 30 s and then the surface was air-dried according to manufacturer's instructions [18, 19].

**Figure 4:**
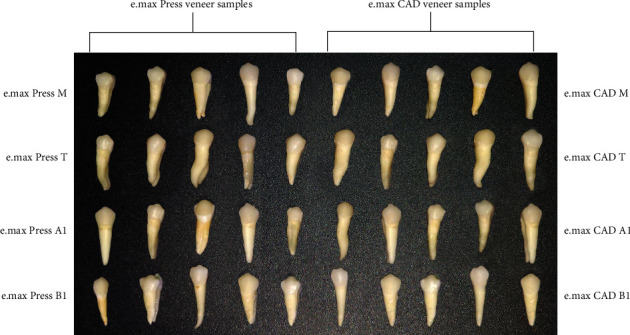
Forty veneer samples divided into eight groups according to material and cement shade. The veneers are cemented to their corresponding teeth for color measurement after cementation.

For cementing the conditioned laminate veneers to their corresponding conditioned teeth, a thin layer of bonding agent (porcelain bonding resin; Choice 2, Bisco) was applied to the inside of each veneer. It was not light-cured and cement was placed over it in accordance with the previously allocated groups. The veneers were gently seated on their corresponding teeth and then light-cured for 3–5 s to tack the veneer in place. The excess cement was then removed, and the veneers were light-cured for 40 s from all surfaces according to manufacturer's instructions, as shown in Figure 4. After cementation all specimens were stored in distilled water at 37°C for 24 h until color measurements were performed [18]. The color measurement was completed after cementation and the colorimeter automatically calculated the *∆E* (delta) value (total color change) according to the following formula:

  
$$\begin{array}{c} \Delta E=\sqrt{\left(\Delta {\text{L}}^{*}\right)}2+\left(\Delta {\text{b}}^{*}\right)2+\left(\Delta {\text{a}}^{*}\right)2, \end{array}$$

where *Δ*L^*∗*^ is the difference of L^*∗*^, *Δ*a^*∗*^ is the difference of a^*∗*^, and *Δ*b^*∗*^ is the difference of b^*∗*^.

The readings were recorded and tabulated according to their groups for further statistical analysis and comparisons where color difference values ≤1 are considered not perceptible by the human eye, while values >1 and ≤3.3 *∆E* units are identifiable by skillful dentists and considered within clinical acceptance, while *∆E* values >3.3 are identified by untrained observers such as patients, and are, therefore, considered unsatisfactory [1, 20–22].

A one-way analysis of variance (ANOVA) and Duncan's post hoc test were used to test the effect of material and cement shade on translucency and their interactions with mean color parameters. The significance level was set at *p* ≤ 0.05. Statistical analysis was performed using International Business Machines (IBM), Statistical Package for the Social Sciences (SPSS) Statistics Version 25 for Windows (IBM, Armonk, NY, USA).

## 3. Results

The distribution of the data was normal after being verified and subjected to normality tests (Kolmogorov–Smirnov and Shapiro–Wilk tests). Descriptive statistics showing means and standard deviations of color change measured in *ΔE* for both ceramic materials cemented with four cement shades to their corresponding teeth are shown in Table 1.

ANOVA was performed after homogeneity of variance and normal distribution had been confirmed. The results showed a significant difference between different ceramic material types (IPS e.max CAD and e.max Press) and the four different cement shades used in this study (Table 2).

**Figure 5:**
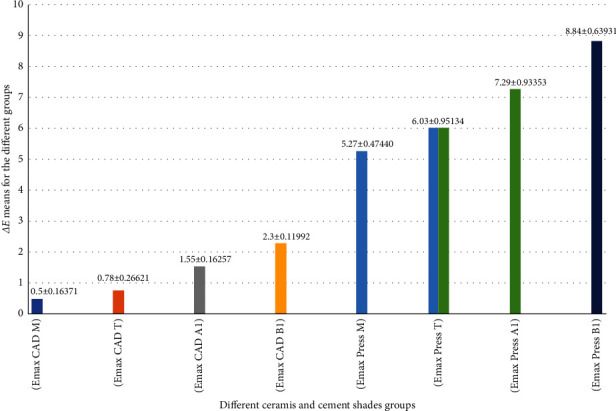
Column graph showing the Duncan's multiple range test results for e.max CAD and e.max Press veneers cemented with different cement shades. The same color indicates no significant difference, different colors indicate significant differences, and two colors indicate no significant difference in both groups for the same color. CAD, computer aided design.

In terms of the differences between different ceramic materials and different cement shades, the post hoc Duncan's multiple range test showed a significant difference in *ΔE* between IPS e.max Press and IPS e.max CAD ceramics for all groups. The highest color change was for the IPS e.max Press M group (8.84 ± 0.63931 *ΔE* units) and the lowest color change for the IPS e.max CAD M group (0.5 ± 0.16371 *ΔE* units). There was a significant difference in *ΔE* among cement shades for both the IPS e.max Press and IPS e.max CAD groups, as shown in Figure 5.

## 4. Discussion

When light is transmitted within a dental restoration, passing through to the surface of the cement, the cement shade can reflect back, thereby affecting the perceived color of the ceramic. Accordingly, resin cement can significantly change the esthetic effect of veneer restorations by masking the underlying tooth color as well as decreasing the translucency of the veneer [23].

Ceramic material types and cement shades significantly affect the final color of a laminate veneer restoration. Therefore, the null hypothesis that there would be no significant difference in ceramic color with different ceramics and shades of resin cement was rejected. Moreover, there were highly significant differences between the two ceramic material types.

The color of each veneer was compared before and after cementation to calculate the overall change in color (*∆E*), which was dependent on human perception of color [1, 20–22]. Moreover, it has been shown that when the *∆E* is 0, the color is perfect; when 0.5–1.5 units, it is very good; when 1–2, it is good; when 2–3.5, it is clinically perceptible; and when >3.5, it is unacceptable [24].

In this in vitro study, results showed that using the IPS e.max Press type of material, the mean *∆E* values were higher than the clinically acceptable values for all tested cement shades, while the IPS e.max CAD laminate veneer material showed lower mean *∆E* values than the clinically acceptable values for all tested cement shades (Figure 5). This indicated higher translucency for IPS e.max CAD because the lower the difference in *∆E* values before and after cementation, the more translucent the material. The results of the present study were consistent with those of other studies reporting that IPS e.max CAD ceramics showed less color changes than those by IPS e.max Press ceramics, this could be attributed to the linear well-organized crystalline structure that was observed with the high transmittance IPS e.max CAD glass ceramics along with a lower marginal discrepancy that could decease light scattering [25, 26]. Also in a study that has shown the contrast ratio (CR) variation among the different LT ceramics for Celtra Duo had the highest level of opaqueness (74.1 ± 1.1) while e.max CAD had the lowest level of opaqueness (71.3 ± 1.1) followed by Press type of material (73 ± 1.5) [27].

Some authors have reported that samples fabricated by computer-aided manufacturing (IPS e.max CAD) have less color changes than IPS e.max Press, also in this study, the greatest color changes were observed for IPS e.max Press [28].

In terms of the effect of resin cement shades, our findings showed that the overall color of IPS e.max CAD and IPS e.max Press laminate veneers was influenced by different shades of resin cement, and there were significant changes in the *∆E* value among the different cement shades, as shown in Figure 5. This finding is consistent with those of other studies that showed significant color changes among the different cement shades for IPS e.max Press and IPS e.max CAD [29–31]. In contrast, this finding is inconsistent with those of other studies that showed that cement shade did not affect overall laminate veneer color and this may be explained by the limited cement thickness (30 μm) [30]. Moreover, different shades of resin cements produced different levels of color changes, and these changes were significant. This finding is consistent with those of many studies that have shown a significant effect of changing shades of resin cement under veneer, even within the clinically acceptable range [9, 10, 24, 32, 33]. The findings of other studies disagree with the present one, stating that changing shades of resin cement under veneer does not have a significant effect on color changes [32–34].

In the current study, for IPS e.max CAD lithium disilicate ceramic, the cement that produced the smallest color changes before and after cementation of laminate veneer and the most excellent color match was the milky bright cement shade (0.5 *∆E* units), followed by 0.78, 1.55, and 2.3 *∆E* units for translucent, A1, and B1 cement shades, respectively. All were within the clinically acceptable level of color change. This finding was consistent with those of other studies that have shown that opaque cement has the smallest *ΔE* value, followed by bleach, transparent, and A1 [35, 36].

For IPS e.max Press lithium disilicate ceramic, the cement that produced the smallest color changes before and after cementation of laminate veneer was also the milky bright cement shade (5.27 *∆E* units), followed by 6.03, 7.29, and 8.84 *∆E* units for translucent, A1, and B1 cement shades, respectively, and all were over the clinically acceptable threshold level of *∆E* ≥3.7 units. This high degree of color change could be due to the high marginal discrepancy and less internal adaptation, which could increase light scattering with the nonuniform thickness of IPS e.max Press samples due the wax pattern and the use of the Press material to construct the veneers. This finding was consistent with that of another study that showed that resin cements can affect the final color of CV restorations (IPS e.max Press) and the extent of this effect varies according to the resin cement shade [20]. There was no significant difference between the A1 cement shade and either translucent or B1 cement shade.

This study had some limitations such as the size of the sample with the lack of oral environmental conditions simulation so further study is needed to evaluate the effect of these factors on the translucency of laminate veneers as there was a difficulty in collecting large number of extracted teeth because of the ethical implications of using animals or humans in research as well as the cost, resources, and time required.

## 5. Conclusions

Different shades of resin cements produce different levels of color changes, and, these changes were significant therefore, different shades of resin cements can help changing the color of the overall veneer to achieve an acceptable color match. Different manufacturing techniques of either emax CAD or emax Press lithium disilicate significantly affect the color changes of CVs. Careful selection of materials and cement shade can minimize color changes in laminate veneers.

## Figures and Tables

**Table 1:** Means and standard deviations of the different groups (e.max CAD and e.max Press) with different cement shades.

Groups	*N*	Mean (*∆E*)	Std. deviation (*∆E*)
IPS e.max CAD cemented with translucent cement shade (e.max CAD T)	5	0.7820	0.26621
IPS e.max CAD cemented with B1 cement shade (e.max CAD B1)	5	2.3060	0.11992
IPS e.max CAD cemented with A1 cement shade (e.max CAD A1)	5	1.5560	0.16257
IPS e.max CAD cemented with milky bright cement shade (e.max CAD M)	5	0.5000	0.16371
IPS e.max Press cemented with A1 cement shade (e.max Press A1)	5	7.2920	0.93353
IPS e.max Press cemented with milky bright cement shade (e.max Press M)	5	5.2720	0.47440
IPS e.max Press cemented with translucent cement shade (e.max press T)	5	6.0300	0.95134
IPS e.max Press cemented with B1 cement shade (e.max Press B1)	5	8.8480	0.63931
Total	40	4.0733	3.10258

Abbreviations: CAD, computer aided design; IPS e.max, lithium disilicate glass–ceramic material (Ivoclar, Schaan/Liechtenstein); Std., standard.

**Table 2:** ANOVA for the eight different groups (e.max CAD and e.max Press) with different cement shades.

ANOVA					
IPS e.max CAD and IPS e.max Press cemented with different cement shades					
	Sum of squares	d*f*	Mean square	*F*	Sig.
Between groups	357.423	7	51.060	90.823	0.000
Within groups	17.990	32	0.562	—	—
Total	375.413	39	—	—	—

Abbreviations: ANOVA, one-way analysis of variance; CAD, computer aided design; IPS e.max, lithium disilicate glass–ceramic material (Ivoclar, Schaan/Liechtenstein).

## Data Availability

All data are available on request, please address all correspondence concerning data availability to Ali Dhahee Malallah at ali.dhahi@uomosul.edu.iq.
